# Use of Hybrid Mineral Filler with High Emissivity in Asphalt Mixture for Cooling Road Pavements

**DOI:** 10.3390/ma16010175

**Published:** 2022-12-25

**Authors:** Lingxiang Kong, Ling Xu, Yinfei Du, Jiao Jin, Giuseppe Loprencipe, Laura Moretti

**Affiliations:** 1 Key Laboratory of Traffic Safety on Track Ministry of Education, School of Traffic & Transportation Engineering, Central South University, Changsha 410075, China; 2 Key Laboratory of Road and Traffic Engineering of Ministry of Education, Tongji University, Shanghai 201804, China; 3 School of Civil Engineering, Central South University, Changsha 410075, China; 4 Key Laboratory of Highway Engineering of Ministry of Education, Changsha University of Science & Technology, Changsha 410114, China; 5 Department of Civil, Constructional and Environmental Engineering, Sapienza University of Rome, Via Eudossiana 18, 00184 Rome, Italy

**Keywords:** asphalt pavement, emissivity, thermal conductivity, temperature reduction, urban heat islands

## Abstract

Road asphalt pavements cover a high percentage of urban size and contribute to heat islands. This study proposed a new method to cool asphalt pavement by incorporating a kind of hybrid mineral filler (HMF) with high emissivity into a reference asphalt mixture prepared with limestone mineral filler (LMF). The physical, emissive, solar reflective, and rheological properties of asphalt mastic and the thermal performances of asphalt mixture were covered to investigate the possibility of the proposed strategy. From Fourier transform infrared spectrum test, it can be found that HMF was physically blended with asphalt. The emissivity results show that HMF increased the emissivity of asphalt mastic from 0.9204 to 0.9820. The asphalt mastic containing HMF had similar solar reflectance with the control one. In addition, HMF could enhance the rutting resistance of asphalt mastic according to the results of multiple stress creep recovery tests. When HMF replaced LMF, the thermal conductivity of the asphalt mixture with HMF increased by 0.26 W/(m·K) (the reference value was 1.72 W/(m·K)). The combined effect of high emissivity and thermal conductivity led to a lower surface temperature (i.e., −5.4 °C) in the tests. The results of this study demonstrate that HMF is a potential material to cool asphalt pavements.

## 1. Introduction

In the literature, previous studies showed that traditional asphalt pavements often have less than 0.1 albedo at the construction [1,2], although it increases during the service life [3,4]. The low albedo makes pavement inclined to absorb more solar radiation than light surfaces [5,6]. Therefore, this property raises the temperature of asphalt pavement [7,8]. According to the scientific literature, the peak surface temperature of asphalt pavement is up to 10 °C higher than conventional cement concrete pavement or other paving materials in the same region and climate conditions [9,10].

The high upper surface temperature of asphalt pavement implies many problems [11,12]. Asphalt pavement releases sensible heat into the atmosphere, which contributes to the formation of the urban heat island effect [13,14]. The asphalt on the surface of high-temperature pavements is easy to experience thermo-oxidative ageing [14,15], which reduces the fatigue-resisting performance of the wearing layer [16,17]. Moreover, asphalt pavements at high surface temperature always have a high inner temperature, which becomes one of the primary factors resulting in rutting [18,19]. It is therefore imperative to propose effective solutions to reduce the temperature of asphalt pavements [13,20].

There are many ways to cope with the high-temperature asphalt pavement, including thermally reflective materials [21,22], open-graded asphalt mixture [4,23], phase change asphalt mixture [24,25], solar energy collecting pavement [4,26], and thermally regulating asphalt pavement [27,28]. These technologies affect the solar reflectance, water evaporation, heat change, and conduction of the asphalt pavement [4,20]. Many studies have shown that emissivity is one of the most important variables affecting the thermal performance of road asphalt pavements. Gui et al. concluded that increasing the emissivity of asphalt pavement by 0.03 helps to reduce the maximum peak temperature by approximately 2 °C [29].

Researchers around the world already investigated the thermal parameters of various road materials and researched to optimize thermal conductivity or thermal diffusivity for cooling pavement technology [10,21,27]. Although the effect of emissivity differences among pavement surfaces with various materials (e.g., cement concrete and grass) on the pavement temperature was also reported [29], by contrast, the related research on the emissivity was relatively limited. It seemed that the potential improvement of the emissivity of asphalt material for cooling pavement technology tended to be neglected because of its black colour. Additionally, the heavy consumption of limestone filler in road construction has caused great pressure on non-renewable abiotic resources [4,23]. It is beneficial to extend the alternative options of fillers for saving mineral resources in the construction industry [30]. Therefore, this research aims to evaluate the possibility of incorporating hybrid mineral filler in asphalt mixture with high emissivity for cooling asphalt pavements. Meanwhile, the high-temperature rheological characteristics of asphalt mastics with hybrid mineral filler were also investigated to promote alternatives for conventional mineral filler.

In this study, a kind of hybrid mineral filler (HMF) with high emissivity was added to asphalt mastics and mixtures by replacing limestone mineral powder (LMF) based on an equal volume concept. Laser particle size analysis, X-ray fluorescence (XRF) spectrophotometer, and X-ray diffraction (XRD) spectrophotometer allowed for analyzing the physical properties of LMF and HMF [31]. The Fourier transform infrared (FTIR) spectrum test was conducted to investigate the interaction effect between asphalt and LMF/HMF [31,32]. The optical properties (e.g., emissivity and solar reflectance) of asphalt mastic and the thermal conductivity and surface temperature characteristic of mixtures have been involved to evaluate the thermal behaviour of the investigated materials. In addition, the rutting resistance of asphalt mastic was investigated using a multiple stress creep recovery (MSCR) test. The high emissivity and thermal conductivity of HMF make it an environment-friendly substitute to LMF in asphalt mixture and a potential material to cool road asphalt pavements.

## 2. Materials and Methods

### 2.1. Materials

#### 2.1.1. Limestone and Hybrid Mineral Fillers

**Figure 1:**
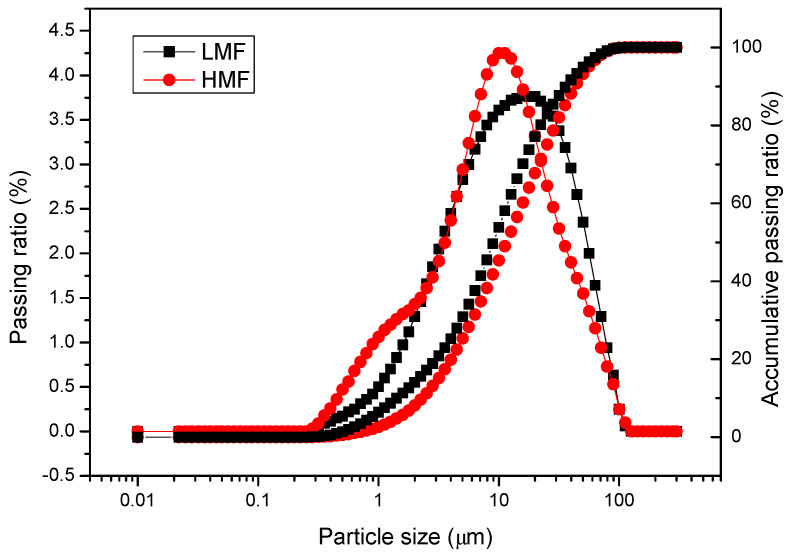
Particle size distributions of LMF and HMF.

LMF was crushed limestone, while HMF was prepared by mixing several mineral raw materials and then sintering them at more than 1200 °C in a waste tailings treatment. The sintering of HMF mainly adopts the hot pressing sintering method before ball milling and mixing. It is a special non-ionic ceramic material that can radiate long-term infrared rays with specific wavenumbers. Figure 1 compares the HMF and LMF particle size distributions.

The particle size distributions of the two fillers were comparable. The entire particle sizes fell in the range of 0.25–102 µm. The maximum passing ratios of LMF and HMF are for 15 mm and 10 mm particle size, respectively. The similar particle size distributions of LMF and HMF ensured the negligible influence of particle size on the investigated performances.

The Rigden void test was conducted to measure the air voids between the compacted particles of fillers. The Rigden void (RV) values of LMF and HMF were 33.47% and 38.38%, respectively. According to AASHTO MP8, both the LMF and HMF satisfied the limit criterion of maximum RV value (50%). HMF with a higher RV value indicated less free asphalt and thus higher mastic stiffness.

The Methylene blue test was carried out according to AASHTO T330 and the Methylene blue values (MBV) of LMF and HMF were 1.24 mg/g and 1.55 mg/g, respectively. Based on the AASHTO standard, the LMF and HMF with an MBV<6 mg/g both lay in an excellent range of performance in asphalt mixture.

**Figure 2:**
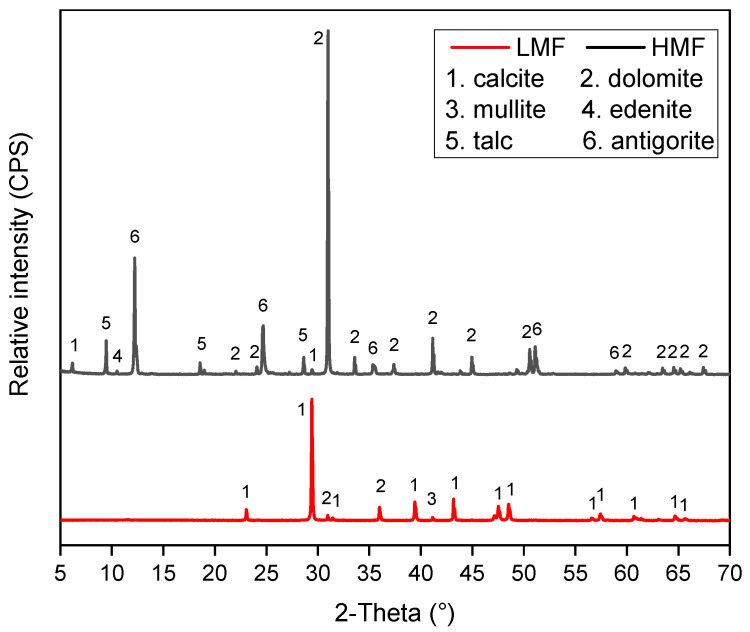
XRD results of LMF and HMF.

The XRD results in Figure 2 highlight the difference in crystal structures between LMF and HMF.

Table 1 lists the chemical compositions of the two fillers determined by XRF.

Figure 2 shows that the LMF used in this study is mainly composed of calcite and dolomite. By contrast, more kinds of mineral phases were identified in the HMF. Given Figure 2 and Table 1, dolomite, antigorite, and talc were the main mineral phases of HMF.

#### 2.1.2. Asphalt Mastic

Asphalt mastic with a constant filler-to-asphalt ratio equal to 1.2 [33,34] was used to characterize the micro, optical, and rheological properties. Five groups of asphalt mastic with different LMF-to-HMF ratios were prepared (Table 2), in which the density of LMF and HMF was 2.78 g/cm^3^ and 2.50 g/cm^3^. Both fillers were added in the mastic based on an equal volume concept.

#### 2.1.3. Asphalt Mixture

An AC-13 asphalt mixture, which was always used in the wearing layer, was used to investigate the effectiveness of HMF as a surface cooler. As for the asphalt mastic, HMF was added by replacing LMF based on an equal volume concept. All the mixtures had an asphalt content of 4.9 wt.%. Three kinds of basalt aggregate with the particle size of 9.5–13.2 mm, 2.36–9.5 mm, and 0–2.36 mm were used. Before testing, the reached void content of all the mixtures was controlled at 4 ± 0.5% to eliminate the uncontrollable influence of void contents. Table 3 lists the specific mass ratios of basalt aggregates, LMF, and HMF in the mixtures. Table 4 lists the aggregate gradation of the control asphalt mixture.

### 2.2. Test Methods

#### 2.2.1. FTIR Spectrum Test

The chemical structure of the asphalt mastics was investigated using an FTIR spectrometer (Nicolet iS50, Thermo Co., Ltd., Waltham, MA, USA). The wavenumber ranged from 650 to 4000 cm^−1^. By comparing the transmittance characteristics of asphalt, HMF, and the asphalt mastic, the interaction effect of asphalt and HMF was distinguished.

#### 2.2.2. Emissivity Test

It was very difficult to measure the emissivity of asphalt mixtures with HMF because the coarse microstructure of the specimen surface had a significant influence on the results [35]. In general, the emissivity of the conventional asphalt pavement was between 0.8 and 0.9 [4]. In this study, the asphalt mastics have been used to characterize the influence of HMF on the emissivity of the asphalt mixtures at room temperature. The samples were prepared by casting hot asphalt mastic on glass slides with a thickness of about 5 mm and cooling them in a drying box. According to the principle of Wien’s displacement law [36], the peak emitted radiation wavelength falls in the range of 8.7–10.6 μm for the materials at 0–60 °C. The law describes the relationship between the peak wavelength (λ_max_) of the blackbody radiation spectrum and its temperature (Equation (1)).

(1) $$\lambda_{\max}=\frac{b}{T}$$

where b is a constant of proportionality called Wien’s displacement constant, equal to 2.898 × 10^−3^ m⋅K, and T is the blackbody temperature in degrees Kelvin.

**Figure 3:**
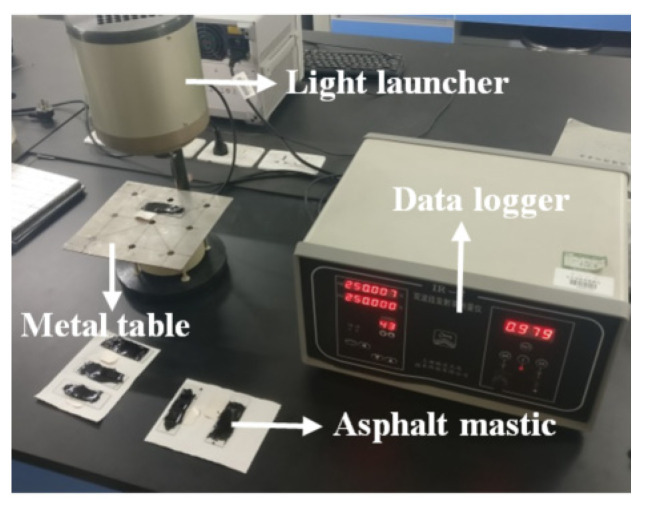
Emissivity measuring instrument.

The emissivity of the samples in the range of 8–14 μm wavelength was measured using an IR-2 dual-band infrared emissivity measuring instrument (Shanghai Photoelectric Technology Co., Ltd., Shanghai, China) (Figure 3). The IR-2 instrument is based on the reflected method by using an active blackbody radiation source to test the normal reflectance of samples, and then convert it into emissivity.

#### 2.2.3. Solar Reflectance Test

Asphalt mastic samples have been used to evaluate the influence of HMF on the solar reflectance of the asphalt mixtures. A UV–VIS–NIR spectrophotometer (Cary 5000, Agilent Technologies (Malaysia) Company) has been used to measure the global (direct + diffuse) spectrum reflectance in the range of 200–2500 nm. The samples were clamped in the integrating sphere to receive light radiation. The specific solar reflectance in the UV–VIS–NIR band was computed according to the ASTM Standard E903-12 [37,38]. It is integrated over the solar spectrum and considers the hemispherical reflectance of solar radiation. The reflection coefficient of asphalt concrete tended to be 0.05–0.2, while the value of cement concrete was 0.21–0.29 [4]. Equation (2) gives the photon energy (E_ph_) for a given wavelength (λ):

(2) $$E_{\mathrm{ph}}=h\cdot c/\lambda$$

where h is the Planck’s constant equal to 6.62608 × 10^−34^ J·s and c is the speed of light in vacuum.

#### 2.2.4. Multiple Stress Creep Recovery (MSCR) Tests

MSCR test was now deemed to be the most reasonable to evaluate the high-temperature deformation resistance of asphalt [39,40,41]. Three stress levels (i.e., 0.1 kPa, 3.2 kPa, and 12.8 kPa [42]) were applied in the MSCR test that was performed using a SmartPave 102 dynamic shear rheometer (Anton Paar Instru., Graz, Austria) at 64 °C. At each stress level, 1 s load duration was followed by recovery at zero load for 9 s. Ten cycles of creep and recovery were applied at each stress level. The non-recovery creep compliance (J_nr_) and the creep recovery rate (R) were used to characterize the deformation resistance of asphalt mastic according to [43].

#### 2.2.5. Thermal Conductivity Test

**Figure 4:**
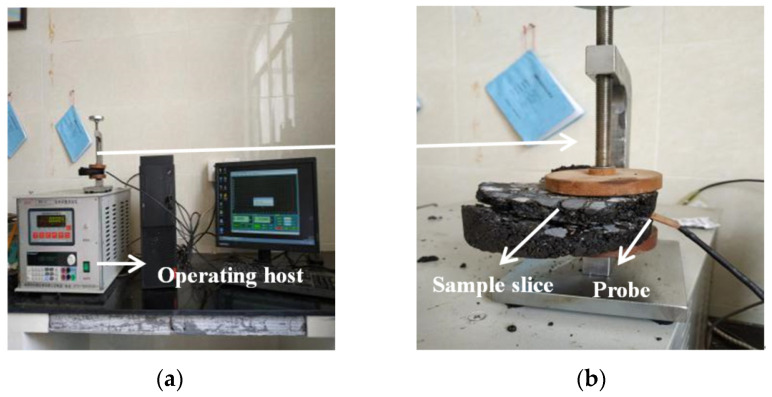
Schematic diagram of thermal conductivity instrument. (**a**) Operating host; (**b**) Measuring sensor.

Each Marshall specimen was cut into several slices with smooth surfaces. A DRE-2C thermal conductivity instrument (Xiangtan Instruments and Meters, Xiangtan, China), which is based on the transient plane heat source method, was used to measure the thermal conductivity (Figure 4a,b). In the test process, a test probe was placed between two sample slices and the two slices were closely connected. The measuring sensor acted both as a temperature sensor for recording the increase of time-dependent temperature and as a heat source to heat the sample of asphalt mixture slices during the test process. Equation (3) allows calculating for the thermal conductivity ($\lambda_{t}$) whose values of asphalt concrete varied from 1.55 to 2.06 W/(m·K) [4].

(3) $$\lambda_{t}=\Phi /\left(-A\frac{\mathrm{dt}}{\mathrm{dx}}\right)$$

where Φ is the heat conduction in W, A is the heat transfer area in m^2^, T is the temperature in K, and x is the coordinate on the heat conduction surface in m.

For each kind of asphalt mixture, more than ten different results were obtained. Their average has been assumed as the measured $\lambda_{t}$.

#### 2.2.6. Indoor Irradiation Test

**Figure 5:**
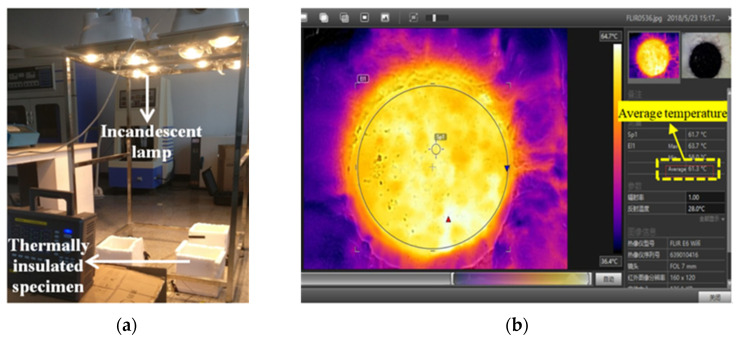
Indoor irradiation test. (**a**) Irradiation test installation; (**b**) Processing of upper surface temperature.

Finally, each Marshall specimen was thermally insulated by spraying foam around it to carry out the irradiation test. Moreover, 275 W incandescent lamps, which were placed over asphalt mixture specimens with a height of 90 cm, were used to simulate the solar radiation (Figure 5a) for over five hours. The temperature profile of the upper surface was recorded by an E6 thermal infrared imager (FLIR Co. Ltd., Wilsonville, OR, USA) with a recording frequency of 6 times per hour. Because of the uneven distribution of upper surface temperature, FILR Tools software [31] allowed the identification of the average temperature (Figure 5b).

## 3. Results and Discussion

### 3.1. FTIR Spectrum

**Figure 6:**
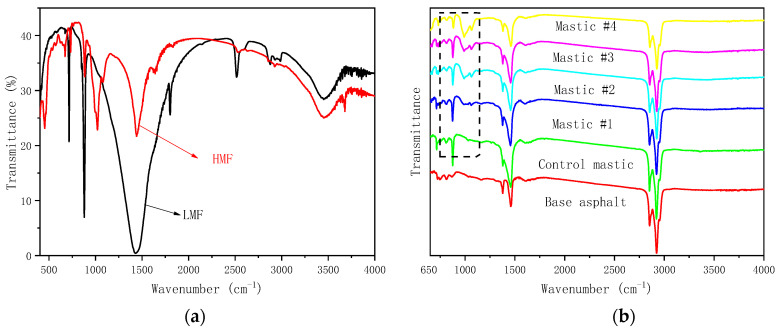
FTIR spectrums. (**a**) LMF and HMF; (**b**) base asphalt and asphalt mastic.

Figure 6a shows the FTIR spectra of LMF and HMF; Figure 6b shows the FTIR spectra of base asphalt and asphalt mastics, respectively.

Figure 6a shows that LMF and HMF had different groups along the wavenumber 650–4000 cm^−1^, indicating that the two powders had different constituents. Figure 6b shows the infrared spectrums of different asphalt mastics. It can be found that the mastics had very similar absorption peaks around the wavenumbers 1380 cm^−1^, 1460 cm^−1^, 2850 cm^−1^, and 2920 cm^−1^, respectively. The mastics with LMF had new absorption peaks around the wavenumbers 700 cm^−1^ and 850 cm^−1^, which depend on the specific absorption characteristic of LMF (Figure 6a). Moreover, the mastics with HMF had new absorption peaks around the wavenumber 1000 cm^−1^ and showed the absorption characteristic of HMF (Figure 6a). There was no new absorption peak which did not cover the infrared spectrums of LMF, HMF, and asphalt, indicating that LMF and HMF were physically blended with asphalt.

### 3.2. Emissivity

**Figure 7:**
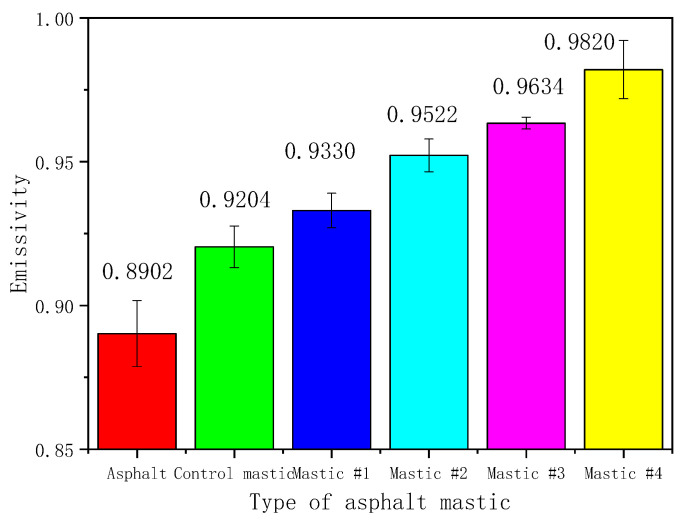
Emissivity results for different asphalt mastics.

Figure 7 shows the results of the measured emissivity values. Error bars are marked to present the measurement uncertainties of tests. Ten replicates were performed in each test. Error bars can be adopted with the STDEVA Function, where standard deviation (SD) and standard error (SE) are calculated in Table 5 according to Equations (4) and (5):

(4) $$\mathrm{SD}=\sqrt{\frac{\sum_{i=1}^{n}{\left(x-\bar{x}\right)}^{2}}{n-1}}$$

(5) $$\mathrm{SE}=\frac{\mathrm{SD}}{\sqrt{n}}$$

where x is the measured variable and $\bar{x}$ is its average value, and n is the number of measurements.

The emissivity of all the asphalt mastics was above 0.92, which was at least 0.03 higher than that of the base asphalt (0.8902). The results reveal that the addition of filler could increase the emissivity of base asphalt. Compared with base asphalt, LMF contributes to a surface coarser, and it can explain part of the testing results because the surface roughness has a significant influence on the emissivity results [35]. To specify the effect of HMF on emissivity, the authors compared the results of the asphalt mastics containing LMF and HMF. When HMF was added to asphalt by replacing an equal volume of LMF, the measured emissivity increased. For example, the emissivity of mastic #4 (i.e., 0.9820) was 0.06 higher than that of the control one (i.e., 0.9204), indicating that HMF improved the emissive property of the asphalt mastic. Therefore, asphalt pavements with HMF can emit more heat into the atmosphere and cool road surfaces.

### 3.3. Solar Reflectance

**Figure 8:**
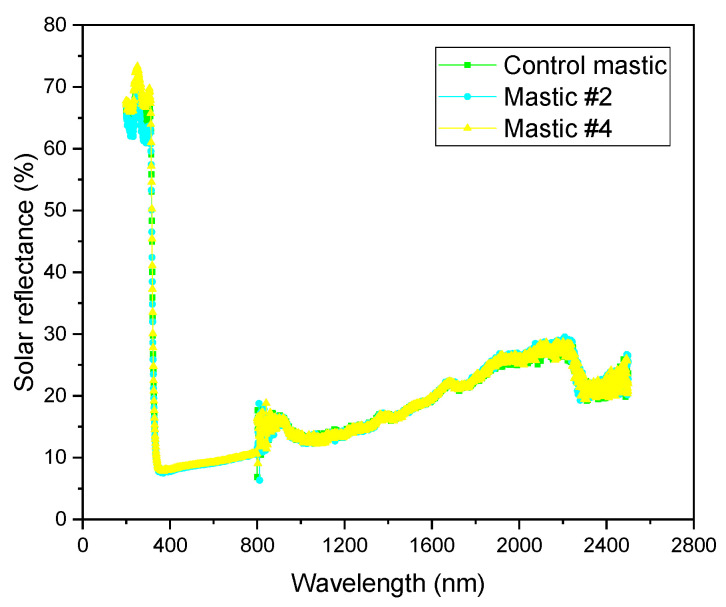
Solar reflectance results of control mastic, mastic #2 and mastic #4.

Three mastics (i.e., control mastic, mastic #2, and mastic #4) were chosen to study the influence of HMF on the solar reflectance; Figure 8 shows their results.

Figure 8 shows that HMF had almost no effect on the reflectance characteristic of asphalt mastic. The results highlight that the solar reflectance of mixtures with LMF or HMF can be overlooked when analyzing the temperature distribution of flexible pavements.

### 3.4. Rutting Resistance Evaluation of Asphalt Mastic

**Figure 9:**
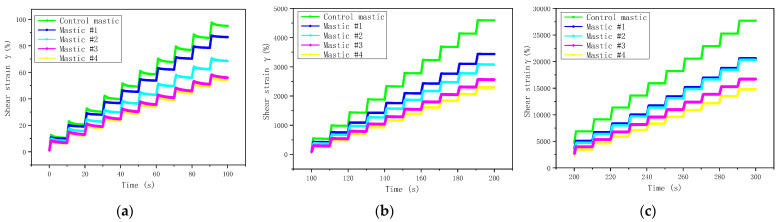
Shear strain variations with time. (**a**) 0.1 kPa; (**b**) 3.2 kPa; (**c**) 12.8 kPa.

The MSCR test was performed to evaluate the rutting resistance of different asphalt mastics. Figure 9a–c show the shear strain variations with time at different stress levels (i.e., 0.1 kPa, 3.2 kPa, and 12.8 kPa, respectively).

**Figure 10:**
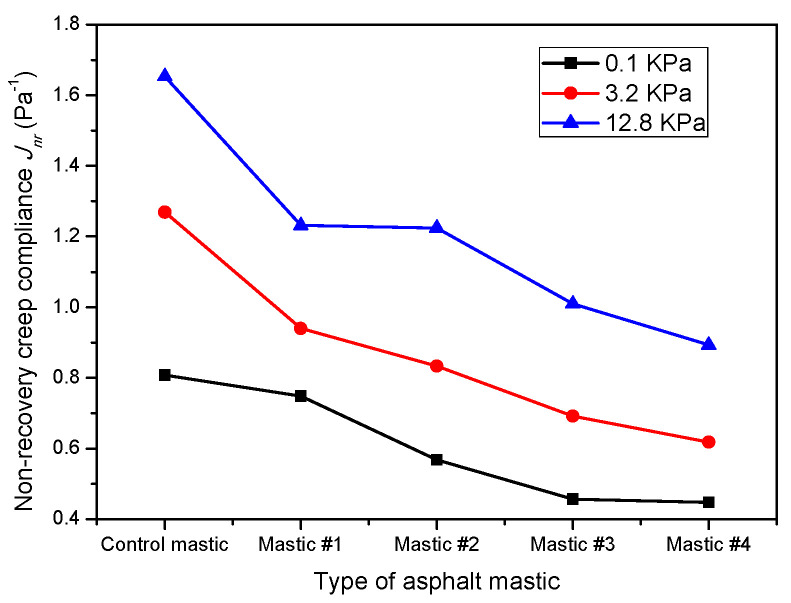
Non-recovery creep compliances.

**Figure 11:**
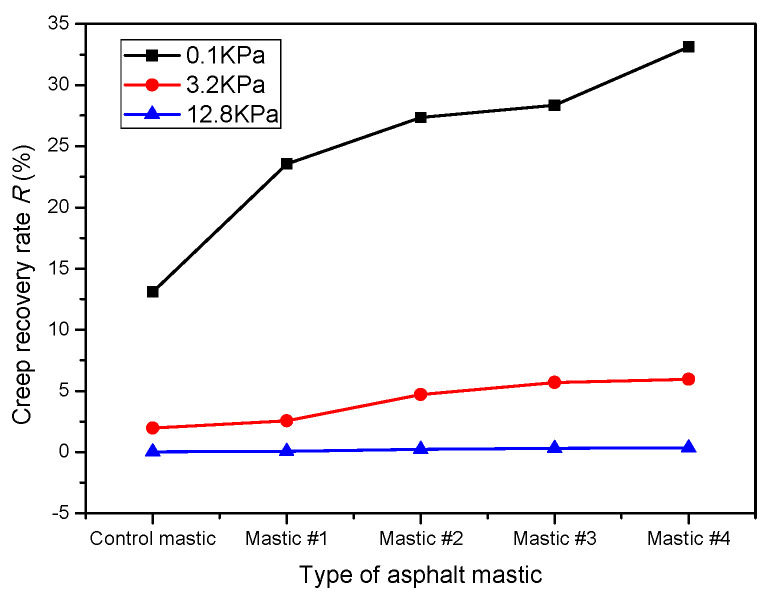
Creep recovery rates.

Due to the same filler-to-asphalt ratio, the shear strain of all the asphalt mastics showed very similar variations with time. However, LMF and HMF had different impacts on the increasing rate of shear strain. According to Figure 9, the non-recovery creep compliance (J_nr_) and the creep recovery rate (R) were used to evaluate the rutting resistance of the tested mastics; Figure 10 and Figure 11 show their values, respectively.

Figure 10 shows that the non-recovery creep compliances of all the asphalt mastics increased with increasing applied stress levels, which was consistent with [44,45]. At the stress level 3.2 kPa, J_nr_ of the control mastic was 1.28 kPa^−1^ and satisfied the heavy (H) level of traffic loading according to AASHTO MP 19-10. Meanwhile, J_nr_ of mastic #4 was 0.62 kPa^−1^ and satisfied the very heavy (V) level of traffic loading. The comparison between J_nr_ values highlights that HMF had a lower ability to increase J_nr_ of asphalt than LMF. That is, HMF could better improve the deformation resistance of asphalt than LMF. Specifically, at the stress level 12.8 kPa, the J_nr,12.8_ values of Mastic #1, #2, #3, and #4 were 25.5%, 25.9%, 39.0%, and 45.9% lower than those of the control mastic, respectively.

The results in Figure 10 and Figure 11 reveal that stress level had a higher influence on R compared with J_nr_. At the stress level of 12.8 kPa, the R of all mastics was nearly equal to 0 and confirmed [46,47,48]. These low R values were related to the considerable strain caused by the high stress [42]. On the other hand, the filler type had an obvious influence on the R at 0.1 kPa and 3.2 kPa stress levels. For example, the R of the control mastic at the stress level of 3.2 kPa was 23.0%, 58.1%, 65.4%, and 67.0% lower than that of Mastic #1, #2, #3, and #4. By contrast, at the stress level of 12.8 kPa, the R of all the asphalt mastics changed in a very small range, indicating that at this stress level, the powder type does not affect the creep recovery rate.

### 3.5. Thermal Conductivity

**Figure 12:**
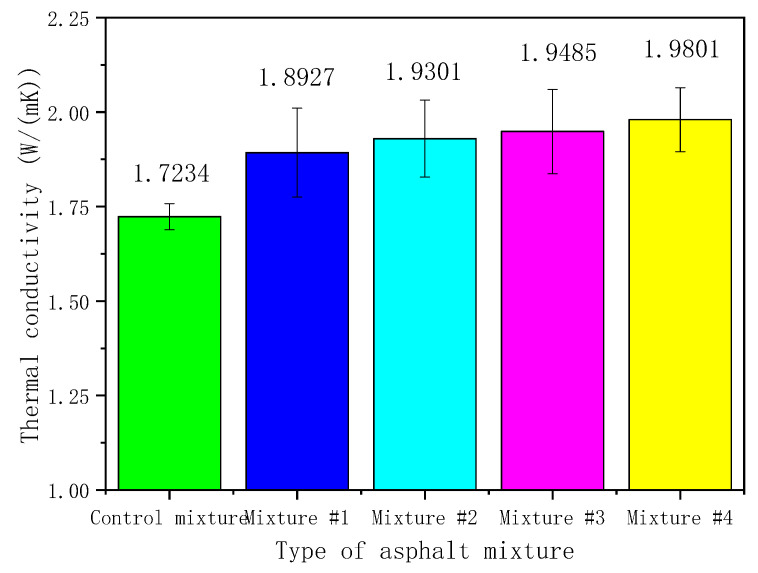
Thermal conductivity of different asphalt mixtures.

To present the influence of HMF on the heat-transfer characteristic of asphalt pavement, the thermal conductivities of asphalt mixtures with different powders were measured. Figure 12 shows the results. Error bars in Table 6 are also marked to present the measurement uncertainties of tests, according to Equations (1) and (2).

Although HMF was added to asphalt mixtures by replacing an equal volume of LMF, the mixtures with HMF had a higher thermal conductivity than that of the control one. According to Figure 12, the average thermal conductivity of Mixture #4 was 0.26 W/(m·K) higher than that of the control one. Meanwhile, a statistical analysis of ANOVA was implemented to determine whether the HMF content had a notable effect on the thermal conductivity. The ANOVA test was conducted with the SPSS software at a 95% significance level (*p* = 0.05), where filler content and thermal conductivity were the independent and dependent variable, respectively. Table 7 shows the addition of HMF exhibited a remarkable effect on the testing results (*p*-value < 0.05).

According to [49,50], high thermal conductivity asphalts for the wearing layer would induce more solar heat to transfer in asphalt pavement, and the upper surface temperature would thus reduce. Then, the temperature distribution of pavements with HMF is validated in the following section.

### 3.6. Temperature Distribution

**Figure 13:**
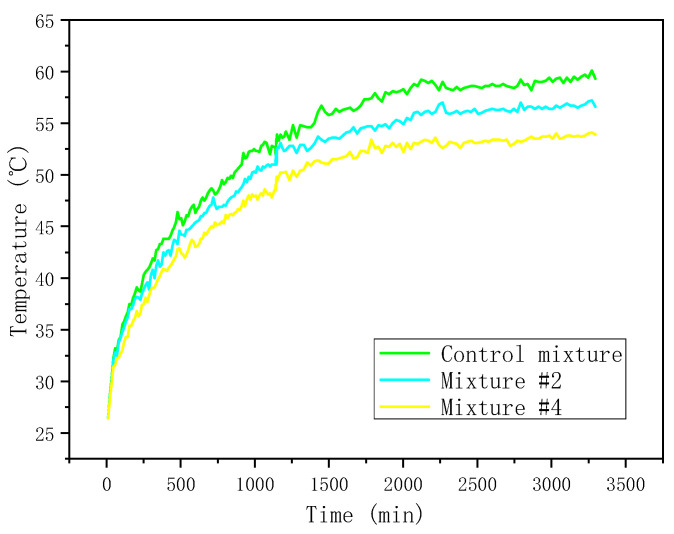
Surface temperature evolution.

To avoid the difference of temperature distribution being too small to be observed, the control asphalt mixture, Mixture #2, and Mixture #4 with 0%, 50%, and 100% HMF were selected. Figure 13 shows the results.

**Figure 14:**
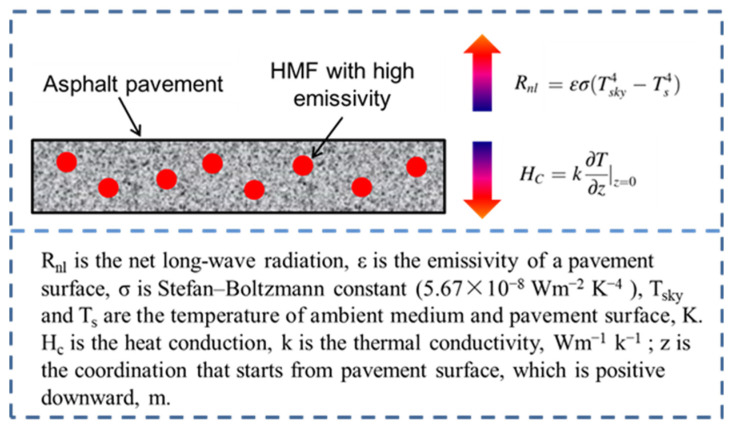
Surface temperature-reducing mechanism of HMF.

Figure 13 shows that the surface temperature of the asphalt mixture specimen with HMF was lower than that of the control one. On the one hand, HMF could increase the emissivity of the asphalt mixture (Figure 7), which resulted in more heat release from the asphalt pavement to the atmosphere and less heat accumulation in the asphalt pavement. On the other hand, HMF enhanced the thermal conductivity of the asphalt mixture (Figure 12), which induced more irradiation heat to be absorbed. Figure 14 explains the surface temperature-reducing mechanism of HMF.

As such, the heat accumulation on the asphalt pavement surface decreased, and the surface temperature thus reduced. The maximum temperature difference between the control mixture and Mixture #4 reached up to 5.4 °C. The result indicates that HMF into asphalt mixtures can limit temperature of pavement surfaces, urban heat islands, and asphalt ageing.

## 4. Conclusions

Road asphalt pavements with thermo-physical properties of high heat absorption tend to intensify the urban heat islands, which causes negative effects on traffic, environment, and health conditions. It has been reported that enhancing the emissivity of asphalt mixtures is a potential way to decrease heat accumulation and thus reduce pavement temperature. This study used HMF with high emissivity to replace LMF in asphalt mixture and investigated the possibility of the above method for cooling asphalt pavement.

- HMF had similar particle distribution, Rigden void, and Methylene blue value with LMF, but had different compositions according to the results of XRD and XRF. It was confirmed that the asphalt mastic with HMF had higher emissivity than that containing LMF;
- By comparing the solar reflectance of the asphalt mastics, it was concluded that HMF had almost no effect on the solar reflectance of the asphalt mixture. From the Fourier transform infrared spectrum test of the asphalt mastic, it can be found that LMF and HMF were both physically blended with asphalt;
- The rutting resisting abilities of the asphalt mastics were evaluated using the MSCR test at the temperature of 64 °C. Both non-recovery creep compliance and creep recovery rate indicate that HMF could enhance the rutting resistance of the asphalt mastic more than LMF;
- The asphalt mixture with HMF presented higher thermal conductivity than that of the control asphalt mixture. The combined effect of high emissivity and thermal conductivity led to up to 5.4 °C lower surface temperature of Mixture #4 than the control one;
- The results in this study indicate that the asphalt mixture with HMF can achieve the effect of cooling asphalt pavement, which can counteract urban heat islands.

For the future study that aims to promote further development and application of HMF in cooling road pavement for heat island mitigation, the following are several recommendations:

1. The surface area of filler and the filler–bitumen relationship should be investigated and established experimentally to guide the further optimization of the filler-to-asphalt ratio;
2. Comprehensive performance tests are suggested in the verification of asphalt mixture performances, including stiffness, resistance to water, rutting, and ageing;
3. In-case experiments with field test section are suggested to verify the in-place temperature reduction effect of HMF on the road pavement surface;
4. An appropriate combination of HMF and other technologies of cooling pavement is recommended for the more efficient mitigation of urban heat islands, such as reflective pavement and evaporative pavement strategies;
5. Life-cycle analysis (LCA) of HMF application on cooling road pavement allows more comprehensive assessments of energy conservation and environment improvement.

## Figures and Tables

**Table 1:** XRF results of LMF and HMF.

Component	Property	SiO_2_	CaO	MgO	Al_2_O_3_	Fe_2_O_3_	Other Oxides	Ignition Loss
LMF	mass	0.196	55.216	0.683	0.087	0.045	0.069	43.704
HMF	ratio (%)	17.740	29.060	25.000	1.590	0.941	0.417	25.252

**Table 2:** Mass ratios of components in asphalt mastic (% by weight).

Mastic Type	Asphalt	LMF	HMF
Control mastic	100	120	0
Mastic #1	100	90	26.9
Mastic #2	100	60	53.9
Mastic #3	100	30	80.9
Mastic #4	100	0	107.9

**Table 3:** Mass ratios of composition in asphalt mixture (% by weight).

Mixture Type	Basalt Aggregates			LMF	HMF
	9.5–13.2 mm	2.36–9.5 mm	0–2.36 mm		
Control mastic	28	27	41	4	0
Mastic #1	28	27	41	3	0.9
Mastic #2	28	27	41	2	1.8
Mastic #3	28	27	41	1	2.7
Mastic #4	28	27	41	0	3.6

**Table 4:** Aggregate gradation for control asphalt mixture.

Sieve size (mm)	16.0	13.2	9.5	4.75	2.36	1.18	0.6	0.3	0.15	0.075
Passing ratio (% by weight)	100	94.6	69.0	43.8	29.7	20.3	14.3	9.1	7.5	6.0

**Table 5:** Error statistics results of emissivity test.

Parameter	Base Asphalt	Control Mastic	Mastic #1	Mastic #2	Mastic #3	Mastic #4
SD	0.0363	0.0229	0.0190	0.0179	0.0065	0.0320
SE	0.0115	0.0072	0.0060	0.0057	0.0021	0.0101

**Table 6:** Error statistics results of the thermal conductivity test.

Parameter	Control Mastic	Mastic #1	Mastic #2	Mastic #3	Mastic #4
SD	0.1103	0.3719	0.3209	0.3534	0.2690
SE	0.0349	0.1176	0.1015	0.1118	0.0851

**Table 7:** ANOVA results of thermal conductivity.

	DF	Adj. SS	Adj. MS	F-Statistic	*p*-Value
Between groups	4	0.396	0.099	1189.384	0.001
Within groups	45	0.004	0.001		
Total	49	0.399			

Note: DF is the degree of freedom, Adj. SS is the adjusted sum of the squares, and Adj. MS is the adjusted mean square.

## Data Availability

The data presented in this study are available on request from the corresponding author.

## Nomenclature

AC	Asphalt concrete
DSR	Dynamic shear rheometer
FTIR	Fourier transform infrared
HMF	Hybrid mineral filler
H_c_	Heat conduction
J_nr_	Non-recoverable creep compliance
k	Thermal conductivity
LCA	Life-cycle analysis
LMF	Limestone mineral filler
MSCR	Multiple stress creep and recovery
R	Creep recovery rate
R_nl_	Net long-wave radiation
SD	Standard deviation
SE	Standard error
T_s_	Temperature of pavement surface
T_sky_	Ambient temperature
UV-VIS–NIR	Ultraviolet-visible-near infrared
XRF	X-ray fluorescence
XRD	X-ray diffraction
